# Progesterone Levels on the Day of Frozen-thawed Embryo Transfer in Artificial Cycles with Vaginal Progesterone Do not Predict Clinical Pregnancy: a Prospective Study

**DOI:** 10.1007/s43032-026-02157-w

**Published:** 2026-07-22

**Authors:** Suelen Maria Parizotto Furlan, Carla Maria Franco Dias, Rui Alberto Ferriani, Paula Andrea Navarro

**Affiliations:** 1 Department of Gynecology and Obstetrics, Ribeirão Preto Medical School, University of São Paulo, Ribeirão Preto, São Paulo Brazil; 2 Embryo Human Reproduction Center/FERTGROUP, Curitiba, Brazil

**Keywords:** Progesterone, Clinical pregnancy, Luteal phase support, Artificial cycle, Frozen-thawed embryo transfer

## Abstract

Some studies have reported that low serum progesterone (P) levels on the day of frozen-thawed embryo transfer (FET) are associated with lower pregnancy rates following artificial endometrial preparation (AEP) cycles. Other studies, however, have found no consistent association between serum P levels and pregnancy outcomes. This prospective cohort study included 235 patients who underwent FET following AEP cycles with estradiol valerate and vaginal micronized P. All patients received either blastocyst- or cleavage-stage embryos derived from their own oocytes, without preimplantation genetic testing for aneuploidies. Blood samples were collected on the day of FET to measure serum P concentrations. Serum P levels on the day of embryo transfer did not differ significantly between patients who achieved CP and those who did not (12.76 ± 4.1 ng/mL vs. 11.9 ± 5.6 ng/mL; *p* = 0.3318). The optimal serum P cutoff level associated with higher CP rates was 10.6 ng/mL, with a sensitivity of 72%, a specificity of 44%, and an AUC of 0.58, indicating poor predictive value. However, patients with serum P levels above this cutoff had a significantly higher CP rate than those with lower values (25.9% vs. 14.6%; *p* = 0.0372). In the multivariate logistic regression model, this difference was not statistically significant (OR = 2; 95% CI: 0.9–5). Routine monitoring of serum P levels on the day of embryo transfer in AEP cycles using vaginal micronized P is not justified, as serum P levels do not effectively predict CP outcomes in cycles using autologous oocytes.

## Introduction

Approximately one in six people worldwide experience infertility at some point in life [30]. To mitigate the negative impacts caused by the inability to conceive—or even to plan the timing, number, and spacing between children—assisted reproductive technologies (ARTs) have emerged as a promising strategy to address this challenge. As reproductive medicine advances, the use of embryo cryopreservation has become increasingly common [15, 22, 24], along with a progressive rise in the number of endometrial preparation cycles with frozen-thawed embryo transfer (FET) [6].

Several factors contribute to the current trend toward “freeze-all” cycles, in which all produced embryos are cryopreserved instead of being transferred during the fresh cycle (Roque et al. 25. Embryo cryopreservation increases the cumulative live birth rate per initiated cycle [24, 29], is cost-effective, and is simpler than performing multiple controlled ovarian stimulation (COS) cycles for fresh embryo transfer [10]. Additionally, it allows for better clinic scheduling, more controlled progesterone exposure [13], and provides greater safety for patients at risk of ovarian hyperstimulation syndrome [9].

In FET, endometrial preparation for cryopreserved embryo transfer can be carried out in several ways: artificial cycle, natural cycle, stimulated natural cycle, or modified natural cycle. In artificial cycles, exogenous hormones are administered to mimic the phases of the menstrual cycle. This approach suppresses endogenous ovarian activity, and no corpus luteum is formed. Conversely, in natural cycles, endometrial preparation occurs through endogenous hormone production, initially from a dominant follicle and subsequently from a corpus luteum after ovulation. The natural cycle may be spontaneous—especially in ovulatory women—or induced (stimulated natural cycle) using oral ovulation inducers or gonadotropins in anovulatory women. In natural cycle preparations, ovulation may be triggered with human chorionic gonadotropin (hCG) in selected cases—a strategy referred to as the modified natural cycle [10, 14].

Artificial endometrial preparation (AEP) cycles are practical, flexible, and widely used in clinical practice. However, the literature does not clearly define the optimal route or dosage of exogenous progesterone administration for luteal phase support in artificial cycles [1, 26]. Similarly, there is no consensus on whether serum P measurement on the day of FET is clinically relevant, nor has it been definitively established whether a specific serum P cutoff on the day of embryo transfer can accurately predict pregnancy success rates. Most studies evaluating the impact of serum P concentrations on the day of FET in artificial cycles are observational and yield conflicting results [7, 12–14, 28, 31]. A systematic review and meta-analysis published by Melo et al. [20] showed that serum P levels below 10 ng/mL in women undergoing endometrial preparation for cryopreserved embryo transfer are associated with lower pregnancy and live birth rates and a higher risk of miscarriage. Nonetheless, the review highlights ongoing uncertainty regarding whether higher P levels improve outcomes, due to heterogeneity among studies, a lack of prospective data, and wide confidence intervals.

Given the limitations in the current literature, it is necessary to determine whether serum P concentration on the day of FET in AEP cycles is a reliable predictor of clinical pregnancy (CP) and, if so, to establish an optimal cutoff value. In this prospective study, the authors evaluated the ideal cutoff point for serum P on the day of FET that correlates with higher CP rates following AEP using vaginal micronized progesterone. Additionally, the study investigated potential factors influencing serum P levels on the day of embryo transfer, as well as variables associated with the occurrence or absence of CP.

## Materials and Methods

### Study Design and Setting

This prospective cohort study was carried out at the Human Reproduction Center of the Ribeirão Preto Clinics Hospital (HCRP). All patients eligible for artificial endometrial preparation (AEP) for frozen-thawed embryo transfer (FET) between November 2021 and June 2023 were invited to participate.

### Study Population

The sample size analyzed was based on a convenience sample, consisting of all eligible patients who underwent an artificial endometrial preparation cycle for warmed embryo transfer between November 2021 and June 2023.

All patients who underwent AEP for FET between November 2021 and June 2023 were invited to participate in the study, provided they met the following inclusion criteria: age under 45 years at the time of ovarian stimulation for *in vitro* fertilization (IVF) and embryo vitrification; age under 50 years at the time of the FET cycle; body mass index (BMI) below 35 kg/m^2^; use of autologous oocytes; and no preimplantation genetic testing for embryo aneuploidy (PGT-A). Eligible patients who agreed to participate signed an informed consent form and were included in the study.

Patients were excluded if the embryo transfer was canceled; if endometrial preparation was performed using dydrogesterone; if blood samples were not collected on the day of FET for P concentration measurement; if accurate P values were unavailable due to sample dilution errors; or if the patients were lost to follow-up.

The number of transferred embryos, whether at the cleavage or blastocyst stage, ranged from one to three.

## Study Protocol

### Data Source and IVF Laboratory

Clinical data from the COS cycle in which the embryos were cryopreserved, as well as from the endometrial preparation cycle for FET, were obtained from the participants’ medical records.

All patients underwent COS to produce embryos for cryopreservation. The choice of COS protocol was based on individual characteristics and ovarian reserve, as well as ovarian response in previous cycles, when applicable (*i.e*., in patients who were not undergoing their first cycle). Most COS cycles were performed using urinary FSH/LH in a GnRH antagonist protocol, with ovulation triggered using urinary or recombinant hCG. In all cases, intracytoplasmic sperm injection (ICSI) was performed following oocyte retrieval, 34 to 36 h after the trigger, in accordance with institutional protocol.

Fertilization, characterized by the presence of two pronuclei and two polar bodies, was assessed 18 to 20 h after ICSI. The fertilization rate was calculated as the number of fertilized oocytes divided by the number of injected oocytes (*i.e*., those submitted to ICSI), multiplied by 100.

Embryos were evaluated for cleavage approximately 44 h (Day 2 post-ICSI, D2) and 66 to 68 h (Day 3, D3) after ICSI. Cleaved embryos were defined as those with two or more cells. The cleavage rate was calculated by dividing the number of cleaved embryos by the number of fertilized oocytes, multiplied by 100.

Cleavage-stage embryos were classified according to the criteria defined by the Istanbul consensus (Alpha Scientists in Reproductive Medicine and ESHRE Special Interest Group of Embryology 2. On D2, embryos with four cells and grade 1 morphology were considered top-quality, on D3, those with eight cells and grade 1 morphology were similarly classified as top-quality. When fewer than three embryos with good morphological quality were obtained, they were cryopreserved at the cleavage stage. If three or more top-quality embryos were available, they were cultured further to the blastocyst stage. In these cases, between 114 and 138 h after ICSI (on fifth or sixth day of development, D5 or D6), the blastocysts were evaluated using the same Istanbul consensus criteria. Those graded 3.1.1 or 4.1.1 were considered top-quality and were cryopreserved at the blastocyst stage (D5 or D6) following extended culture.

### Endometrial Preparation Protocol

For endometrial preparation prior to FET, oral estradiol (Primogyna® or Estrofem®, 2.0 mg orally every 8 h) or transdermal estradiol (Estradot® 100 mcg/day, Oestrogel® 4 puffs/day, or Sandrena® gel 3.0 g/day) was initiated between the first and fifth day of the menstrual cycle and administered for 8 to 12 days. Subsequently, transvaginal ultrasound (TVUS) was performed to assess endometrial thickness and pattern, as well as to check for follicular growth.

When the endometrium reached a thickness of ≥ 7 mm and exhibited a trilaminar appearance, vaginal micronized progesterone (Utrogestan® 600 mg/day) was initiated and continued alongside estradiol for two days (for embryos vitrified on Day 2 of development), three days (Day 3 embryos), five days (Day 5 embryos), or six days (Day 6 embryos), until FET was performed. In each patient’s first endometrial preparation cycle, oral estradiol was used initially; if the endometrium failed to reach ≥ 7 mm, the cycle was canceled and a new cycle was scheduled using transdermal estradiol.

All embryo transfers were performed under abdominal ultrasound guidance (Voluson S8, GE HealthCare, SP, Brazil).

### Blood Samples and Hormone Measurement

Peripheral blood samples were collected by the nursing team at the HCRP Human Reproduction Center to measure serum P levels on the day of embryo transfer, between 8:00 and 10:00 a.m. All patients were instructed to administer micronized progesterone on the morning of the transfer, around 7:00–8:00 a.m.

Serum P concentrations were measured using an immunoassay based on direct chemiluminescence technology (Atellica IM PRGE®), performed on the Atellica IM Analyzer system (Siemens Healthineers, SP, Brazil). The assay detection range was 0.21–60 ng/mL (0.67–190.89 nmol/L). Intra-laboratory precision was ≤ 0.13 ng/mL (0.41 nmol/L) of standard deviation for samples < 1.00 ng/mL. The coefficient of variation (CV) was ≤ 13% for samples between 1.00–6.50 ng/mL and ≤ 6% for samples > 6.60 ng/mL. The analytical sensitivity of the assay was 0.10 ng/mL (0.32 nmol/L).

### Follow-up

Patients were followed up until undergoing measurement of the serum beta subunit of human chorionic gonadotropin (β-hCG), performed fourteen days after embryo transfer. Those with a positive β-hCG result were subsequently monitored until TVUS was performed 2 to 3 weeks after the pregnancy test to confirm CP.

### Definitions

Biochemical pregnancy was defined as a positive β-hCG result, measured 14 days after embryo transfer. The biochemical pregnancy rate was calculated by dividing the number of patients with a positive β-hCG result on the 14th day after FET by the total number of FETs performed during the study period, multiplied by 100.

Clinical pregnancy was defined as the presence of an intrauterine gestational sac on the first TVUS, performed 2 to 3 weeks after the β-hCG test. The CP rate was calculated by dividing the number of confirmed clinical pregnancies by the total number of FETs, multiplied by 100.

Low ovarian reserve was defined as the presence of fewer than seven antral follicles (measuring 2–9 mm) at the start of COS in the IVF cycle in which the embryos for FET were generated.

The presence of a urological procedure was determined when sperm retrieval for ICSI involved PESA (percutaneous epididymal sperm aspiration), TESE (testicular sperm extraction), or micro-TESE (microsurgical testicular sperm extraction).

A FET was considered difficult when it required dilation of the external cervical canal or the use of a rigid catheter.

Recurrent gestational loss was defined as two or more previous miscarriages occurring between 6 and 24 weeks of gestation prior to FET.

Recurrent implantation failure, in turn, was defined as three or more previous embryo transfers involving embryos with good morphology and negative β-hCG results.

#### Study Size

A convenience sample comprising all eligible patients who underwent FET in an AEP cycle using estrogen and vaginal micronized P between November 2021 and June 2023 was analyzed.

#### Confounding Factors

The potential quantitative confounding variables analyzed were: advanced age at the time of COS (over 35 years); obesity (BMI ≥ 30 kg/m^2^); and the number of embryos transferred (with higher pregnancy rates observed when two or three embryos were transferred compared to a single embryo).

Meanwhile, the potential qualitative confounding variables analyzed included: presence of adenomyosis; history of recurrent gestational loss; history of recurrent implantation failure; performance of a fresh embryo transfer in the same IVF cycle that generated the embryos (which may imply that the best-quality embryos were transferred during the fresh cycle); transfer of a top-quality embryo in the FET; embryo developmental stage (cleavage-stage embryos—D2 or D3—are associated with lower pregnancy rates than blastocysts); and technically difficult FET.

All potential confounding factors were evaluated, and the statistical analyses were adjusted for the identified confounders.

#### Quantitative Variables

Progesterone concentration on the day of embryo transfer was analyzed as a continuous variable. A Receiver Operating Characteristic (ROC) curve was constructed to determine the cutoff value of P concentration associated with a higher CP rate. This cutoff was subsequently used as a categorical variable in further analyses.

Body mass index (BMI) was treated both as a continuous and categorical variable, and was classified as follows: underweight (BMI < 18.5 kg/m^2^), healthy weight (BMI 18.5–24.9 kg/m^2^), overweight (BMI 25–29.9 kg/m^2^), and obesity (BMI ≥ 30 kg/m^2^).

### Statistical Analysis

All data were collected and recorded by the researchers using a structured data collection instrument. The information was entered into an electronic spreadsheet (Microsoft Excel), verified for accuracy, and subsequently exported to SAS software, version 9.4, for statistical analysis. Quantitative variables were summarized using measures of central tendency and dispersion, while qualitative variables were presented as absolute and relative frequencies.

In order to compare parametric quantitative variables according to the CP outcome, Student’s *t*-test was applied. Data distribution was assessed using histogram plots. The chi-square test was used to evaluate associations between qualitative variables and the CP outcome.

A ROC curve was constructed to identify the optimal cutoff value of serum P concentration associated with CP.

A multivariate logistic regression model was developed to identify which exploratory variables were predictive of the CP outcome. This model estimates effect size measures (odds ratios), which quantify the association between the exploratory variables and the outcome. Additionally, a separate multivariate logistic regression model was constructed to determine which exploratory variables were associated with serum P concentrations above the cutoff value identified by the ROC curve.

## Results

### Participants

A total of 381 patients were treated at the Human Reproduction Center of the Clinics Hospital of Ribeirão Preto Medical School – USP for FET cycles between November 2021 and June 2023. Of these, 58 patients were deemed ineligible due to undergoing a natural endometrial preparation cycle (*n* = 39), undergoing preimplantation genetic testing (*n* = 11), or undergoing an oocyte donation cycle (*n* = 8). Thus, 323 patients were considered eligible and agreed to participate in the study.

**Fig. 1:**
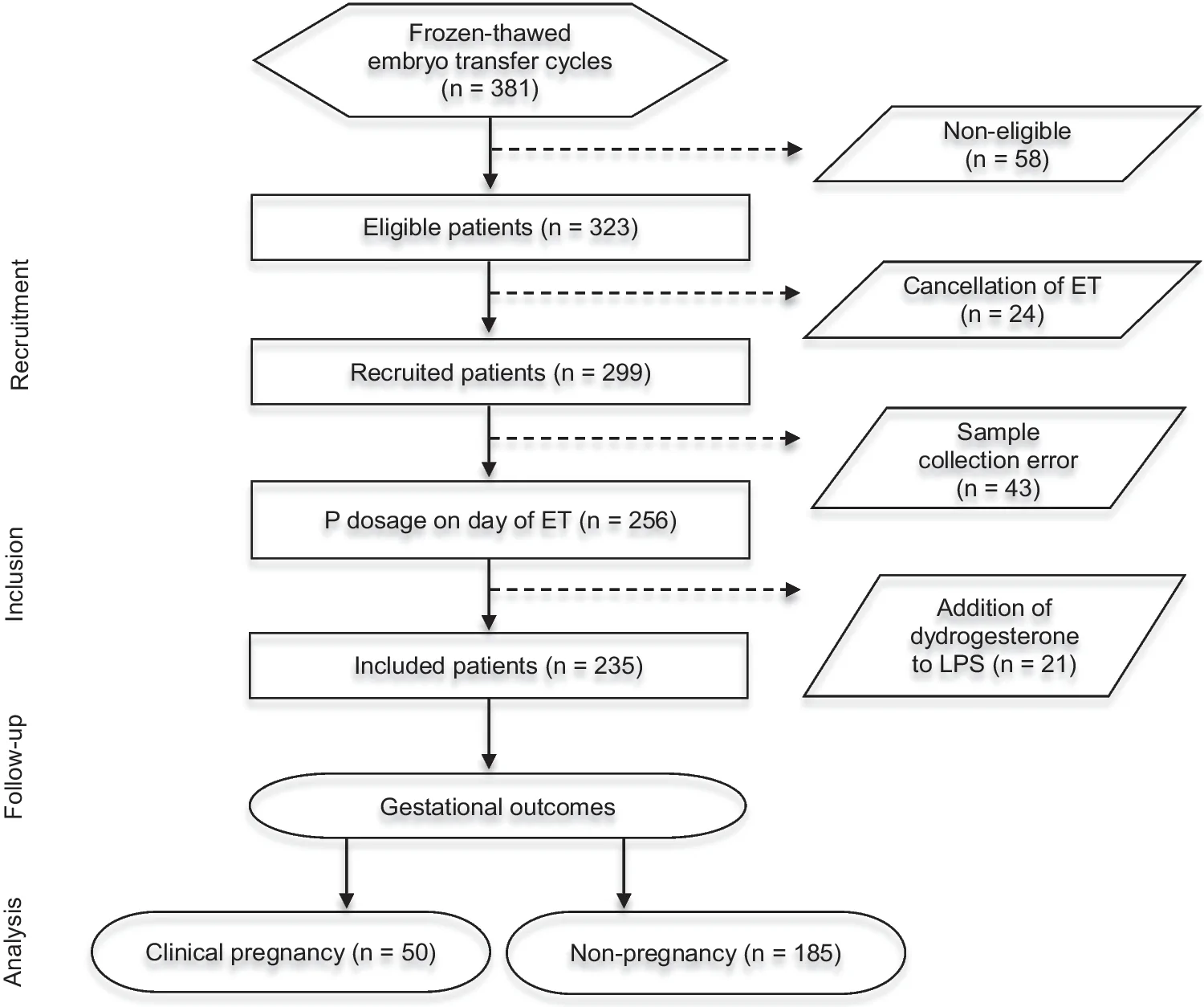
Flowchart illustrating the inclusion and exclusion criteria adopted in this study. *Note: *ET* embryo transfer; *P* progesterone; *LPS* luteal phase support

During the endometrial preparation cycle, 24 patients were excluded for the following reasons: inadequate endometrial response to preparation (*n* = 17), personal reasons (*n* = 3), identification of an endometrial polyp during preparation (*n* = 1), early elevation of serum P levels (*n* = 1), loss of follow-up (*n* = 1), and embryo degeneration during thawing (*n* = 1). On the day of embryo transfer, P measurements were not obtained for 43 patients due to blood collection errors, which included patients who did not have blood collected on the day of frozen embryo transfer (FET) due to logistical constraints at the human reproductive center (*n* = 37) or issues with dilution and laboratory processing (*n* = 6). Following embryo transfer, 21 patients were excluded because the attending physician added dydrogesterone to the luteal phase support (*n* = 21). Ultimately, 235 patients were included and followed until the gestational outcome (Fig. 1).

### Descriptive Analysis Based on Clinical Pregnancy Outcome

A total of 235 patients were evaluated. The mean age on the day of the FET was 38 ± 4 years, and the mean BMI was 25.4 ± 4.44 kg/m^2^. A single embryo was transferred in 43.4% of cases (*n* = 102), two embryos in 55.3% (*n* = 130), and three embryos in 1.3% (*n* = 3). Overall, the CP rate was 21.2% (*n* = 50), and the biochemical pregnancy rate was 30.2% (*n* = 71).

The number of embryo transfers performed at the cleavage stage was 152, whereas 83 transfers were conducted using blastocyst-stage embryos. The clinical pregnancy rate for embryo transfer at the cleavage stage was 14.4% (*n* = 22), and the clinical pregnancy rate for embryo transfer at the blastocyst stage was 33.7% (*n* = 28).

When comparing patients who achieved CP with those who did not, it was observed that the age at the time of COS and FET was significantly higher in the group without CP (Table 1), as was the presence of endometriosis (Table 2). On the other hand, the number of retrieved and fertilized oocytes, cleaved embryos, formed embryos with good morphology, the proportion of top-quality embryos transferred, and the blastocyst transfer rate were significantly higher in the group that achieved CP (Tables 1 and 2).

**Table 1:** Quantitative variables related to patient characteristics, ovarian stimulation cycles for ICSI, and endometrial preparation cycles for frozen-thawed embryo transfer

	Clinical pregnancy YES (*n* = 50)	Clinical pregnancy NO (*n* = 185)		
	Mean ± SD	Mean ± SD	*p*-value	95% CI
No. of patients	50 (21.3%)	185 (78.7%)	**-**	**-**
Age on day of COS (years)	33.6 ± 4.3	35.4 ± 3.6	**0.0024**	**0.6 – 3**
Age on day of FET (years)	35.1 ± 4.8	36.7 ± 3.7	**0.0106**	**0.38 – 2.8**
BMI (kg/m^2^)	25.5 ± 4.4	25.3 ± 4.4	0.7526	-1.6 – 1.1
Duration of infertility (years)	8.2 ± 5.1	8.1 ± 4.2	0.9119	-1.5 – 1.3
Gonadotropin dose (units)	2333.8 ± 836.4	2538.5 ± 699	0.1242	-56.7 – 466.3
Duration of COS (days)	10 ± 2.1	10 ± 1.8	0.8837	-0.5 – 0.6
No. of retrieved oocytes	13.7 ± 6.9	11.4 ± 6.4	**0.0316**	**-4.3 – -0.2**
No. of mature oocytes	10.8 ± 5.8	8.7 ± 5.4	0.1773	-3.8 – -0.3
No. of fertilized oocytes	8 ± 2.9	6.4 ± 3.4	**0.004**	-**2.6 – -0.5**
Fertilization rate (%)	82.8 ± 16.4	79.9 ± 17	0.2659	-8.3 – 2.3
No. of cleaved embryos	7.5 ± 2.7	6.1 ± 3.3	**0.0067**	**-2.4 – -0.3**
Cleavage rate (%)	93.9 ± 9.7	94.4 ± 12.7	0.8068	-3.3 – 4.2
No. of formed embryos	5.2 ± 2.1	4.4 ± 2	**0.0062**	**-1.5 – -0.2**
No. of good-morphology embryos formed	3 ± 1.7	2.2 ± 1.8	**0.0032**	-**1.4 – -0.2**
Days of estradiol use	10.2 ± 2.1	9.9 ± 2.3	0.4166	-1.0 – 0.4
Endometrial thickness on the day of FET (mm)	8.7 ± 1.5	8.7 ± 1.6	0.9928	-0.5 – 0.5
No. of transferred embryos	1.5 ± 0.5	1.6 ± 0.5	0.5541	-0.1 – 0.2
P concentration on the day of FET (ng/mL)	12.76 ± 4.15	11.9 ± 5.69	0.3318	-2.5 – 0.8

*No.* Number; *COS* Controlled ovarian stimulation; *FET* Frozen-thawed embryo transfer; *BMI* Body mass index

Student’s t-test was used to compare quantitative variables

Bold values indicate statistically significant differences between groups (*P* < 0.05)

**Table 2:** Qualitative variables related to patient characteristics, ovarian stimulation cycles for ICSI, and endometrial preparation cycles for frozen-thawed embryo transfer

	Clinical pregnancy YES	Clinical pregnancy NO	*p*-value
No. of patients	50 (21.3%)	185 (78.7%)	-
BMI categorization			
Underweight (BMI < 18)	2 (4%)	5 (2.7%)	
Normal BMI (BMI 18 – 24.9)	17 (34.7%)	89 (48.1%)	0.3657
Overweight (BMI 25 – 29.9)	21 (42.9%)	59 (31.9%)	
Obesity (BMI ≥ 30)	9 (18.4%)	32 (17.3%)	
Type of infertility			
Primary	39 (78%)	138 (74.6%)	0.6202
Secondary	11 (22%)	47 (25.4%)	
Cause of infertility			
Male factor	25 (50%)	108 (58.4%)	0.2889
Endometriosis	4 (8%)	44 (23.8%)	**0.0140**
Tubal factor	10 (20%)	49 (26.5%)	0.3480
PCOS	15 (30%)	35 (18.9%)	0.0894
UI	6 (12%)	20 (10.8%)	0.8120
Adenomyosis	5 (10%)	15 (8.1%)	0.6706
Recurrent gestational loss	2 (4%)	10 (5.4%)	0.6887
Recurrent implantation failure	3 (6%)	26 (14%)	0.1245
Urological procedure	4 (8%)	18 (9.72%)	0.2289
Low reserve	1 (2%)	6 (3.24%)	0.6464
Underwent fresh ET	13 (26%)	38 (20.5%)	0.4060
Estradiol route			
Oral	40 (80%)	149 (80.5%)	
Transdermal	5 (10%)	25 (13.5%)	0.5137
Oral + transdermal	5 (10%)	11 (6%)	
Transfer of good-morphology embryo	39 (78%)	95 (51.3%)	**0.0007**
Embryo stage			
Cleavage	22 (44%)	130 (70.3%)	**0.0006**
Blastocyst	28 (56%)	55 (29.7%)	
Difficult FET	2 (4%)	3 (1.62%)	0.3011

*BMI* Body mass index; *PCOS* Polycystic ovary syndrome; *UI* Unexplained infertility; *ET* Embryo transfer; *FET* Frozen-thawed embryo transfer

The chi-square test was used to compare qualitative variables

Bold values indicate statistically significant differences between groups (*P* < 0.05)

No statistically significant difference was observed in serum P levels on the day of FET between the CP and non-pregnancy groups (Table 1).

### Progesterone Level Cutoff Point Associated with Better Gestational Outcomes

The mean serum P level measured on the day of FET was 12.11 ± 5.4 ng/mL. There was no statistically significant difference in serum P concentrations on the day of FET between the group of patients who achieved CP (12.76 ± 4.15 ng/mL) and those who did not (11.9 ± 5.69 ng/mL).

**Fig. 2:**
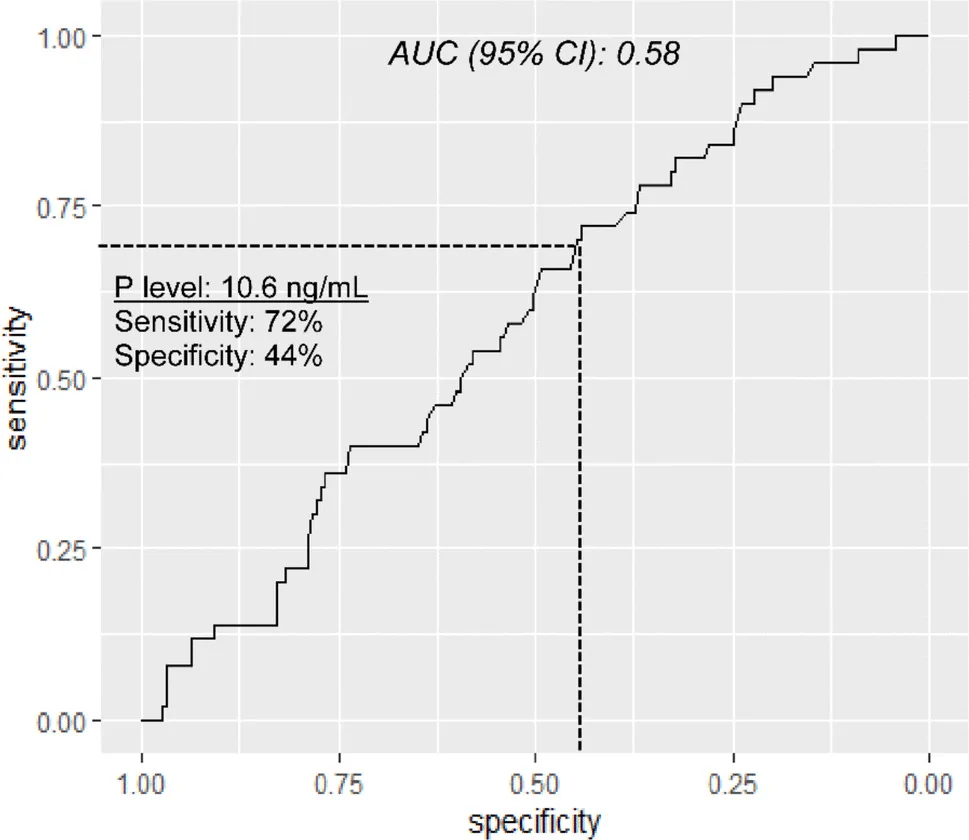
ROC curve of serum P concentration on the day of frozen-thawed embryo transfer following artificial endometrial preparation cycles in relation to clinical pregnancy

The cutoff point for serum P concentration on the day of FET, determined by the ROC curve, was 10.6 ng/mL. Levels below this cutoff were associated with a lower chance of CP, with a specificity of 44%, a sensitivity of 72%, and an area under the curve (AUC) of 0.5826 (Fig. 2).

### Exploratory Analysis of Factors Associated with Clinical Pregnancy Rates on the Day of Embryo Transfer

When performing the univariate analysis, we found a statistically significant difference between the two groups for the qualitative variables: presence of endometriosis, transfer of good-morphology embryo, and embryo stage at transfer. We noted that these three variables impact the CP rate in artificial cycles and remain statistically significant when multivariate logistic regression is applied. In contrast, P levels above 10.6 ng/mL on the day of FET were not predictive of CP (Table 3).

**Table 3:** Multivariate analysis of predictors of clinical pregnancy

	Adjusted odds ratio (95% CI)
Presence of endometriosis	0.2 (0.09 – 0.9)
Age on the day of FET (years)	0.9 (0.7 – 1.2)
Age on the day of COS (years)	0.9 (0.7 – 1.1)
No. of retrieved oocytes	1 (0.9 – 1)
No. of fertilized oocytes	1.1 (0.7 – 0.4)
No. of cleaved embryos	0.7 (0.4 – 1.1)
No. of embryos formed	1.5 (1.1 – 2.1)
No. of good-morphology embryos formed	0.8 (0.6 – 1.2)
Transfer of good-morphology embryo	2.6 (1 – 6.6)
Blastocyst transfer	5.2 (1.9 – 14)
*P* > 10.6 ng/mL on the day of FET	1.9 (0.9 – 4.2)

*FET* Frozen-thawed embryo transfer; *COS* Controlled ovarian stimulation; *P* Progesterone

Odds ratio estimated using the multivariate logistic regression model, in which the event is coded as 0

### Descriptive Analysis According to Serum P Levels on the Day of Embryo Transfer

Statistical analysis was performed based on the serum P cutoff value of 10.6 ng/mL on the day of FET in artificial cycles. Among all patients in the study, 139 (59.1%) had serum P levels above 10.6 ng/mL on the day of FET, while 96 (40.9%) had levels equal to or below this threshold.

The number of retrieved and mature oocytes, the rate of secondary infertility, and the transfer of good-morphology embryos were significantly higher in the group of patients with serum P concentrations above 10.6 ng/mL on the day of FET. Additionally, the CP rate was significantly higher among these patients (Tables 4 and 5).

**Table 4:** Quantitative variables related to patient characteristics, ovarian stimulation cycles for ICSI, and endometrial preparation cycles for frozen-thawed embryo transfer according to the serum P concentration cutoff point on the day of frozen-thawed embryo transfer

	*P* ≤ 10.6 ng/mL	*P* > 10.6 ng/mL	*p*-value	95% CI
No. of patients	96 (40.9%)	139 (59.1%)	-	-
Age on the day of COS (years)	35.1 ± 4	34.9 ± 3.8	0.7975	-1.1 – 0.8
Age on the day of FET (years)	36.3 ± 4.1	36.3 ± 3.9	0.9642	-1 – 1
BMI (kg/m^2^)	25.4 ± 4.3	25.3 ± 4.5	0.8569	-1.2 – 1
Duration of infertility (years)	8.6 ± 3.9	7.8 ± 4.6	0.1847	-1.9 – 0.3
Gonadotropin dose (units)	2516.2 ± 695.5	2487.8 ± 755.6	0.7881	-236.8 – 179.9
Duration of COS (days)	9.8 ± 1.9	10.2 ± 1.9	0.1068	0.08 – 0.9
No. of retrieved oocytes	10.8 ± 5.3	12.7 ± 7.3	**0.0274**	**0.2 – 3.6**
No. of mature oocytes	8 ± 4	9.8 ± 6.2	**0.0107**	**0.4 – 3.3**
No. of fertilized oocytes	6.4 ± 2.9	7 ± 3.6	0.1566	-0.2 – 1.5
Fertilization rate (%)	81.8 ± 15.4	79.6 ± 17.8	0.3299	-6.6 – 2.2
No. of cleaved embryos	6 ± 2.8	6.6 ± 3.5	0.1222	-0.1 – 1.5
Cleavage rate (%)	94.7 ± 13.2	94 ± 11.2	0.6603	-3.8 – 2.4
No. of embryos formed	4.4 ± 1.9	4.6 ± 2.1	0.3501	-0.2 – 0.7
No. of good-morphology embryos formed	2 ± 1.7	2.6 ± 1.9	0.0702	-0.03 – 0.9
Days of estradiol use	10.1 ± 2.3	9.9 ± 2.3	0.4344	-0.8 – 0.3
Endometrial thickness on the day of FET (mm)	8.7 ± 1.7	8.6 ± 1.5	0.7403	-0.5 – 0.3
No. of embryos transferred	1.6 ± 0.5	1.6 ± 0.5	0.9104	-0.1 – 0.1
P concentration on the day of FET	8 ± 1.8	14.9 ± 5.2	**0.0001**	**5.7 – 7.9**

*No.* Number; *COS* Controlled ovarian stimulation; *FET* Frozen-thawed embryo transfer; *BMI* Body mass index

Student’s t-test was used to compare quantitative variables

Bold values indicate statistically significant differences between groups (*P* < 0.05)

**Table 5:** Qualitative variables related to patient characteristics, ovarian stimulation cycles for ICSI, and endometrial preparation cycles for frozen-thawed embryo transfer according to the serum P cutoff value on the day of embryo transfer

	*P* ≤ 10.6 ng/mL	*P* > 10.6 ng/mL	*p*-value
No. of patients	96 (40.9%)	139 (59.1%)	-
BMI categorization			
Underweight (BMI < 18)	2 (2%)	5 (3.6%)	
Normal BMI (BMI 18 – 24.9)	42 (43.8%)	64 (46.4%)	0.7710
Overweight (BMI 25 – 29.9)	36 (37.5%)	44 (31.9%)	
Obesity (BMI ≥ 30)	16 (16.7%)	25 (18.1%)	
Type of infertility			
Primary	81 (84.4%)	96 (69%)	**0.0075**
Secondary	15 (15.6%)	43 (31%)	
Cause of infertility			
Male factor	58 (60.4%)	75 (54%)	0.3260
Endometriosis	19 (19.8%)	29 (20.9%)	0.8412
Tubal factor	25 (26%)	34 (24.5%)	0.7835
PCOS	20 (20.8%)	30 (21.6%)	0.8903
UI	10 (10.4%)	16 (11.5%)	0.7927
Adenomyosis	6 (6.3%)	14 (10%)	0.3020
Recurrent gestational loss	6 (6.3%)	6 (4.3%)	0.5081
Recurrent implantation failure	10 (10.4%)	19 (13.7%)	0.4562
Urological procedure	8 (8.3%)	14 (10%)	0.1134
Low reserve	2 (2%)	5 (3.6%)	0.5022
Underwent fresh ET	23 (24%)	28 (20.1%)	0.4856
Estradiol route			
Oral	78 (81.2%)	111 (78.9%)	
Transdermal	11 (11.5%)	19 (13.6%)	0.8664
Oral + transdermal	7 (7.3%)	9 (6.5%)	
Transfer of good-morphology embryo	46 (48%)	88 (63.3%)	**0.0191**
Embryo stage			
Cleavage	65 (67.7%)	87 (62.6%)	0.4197
Blastocyst	31 (32.3%)	52 (37.4%)	
Difficult FET	2 (2%)	3 (2.1%)	0.9688
Clinical pregnancy	14 (14.6%)	36 (25.9%)	**0.0372**

*No.* Number; *BMI* Body mass index; *PCOS* Polycystic ovary syndrome; *UI* Unexplained infertility; *ET* Embryo transfer; *FET* Frozen-thawed embryo transfer

The chi-square test was used to compare qualitative variables

Bold values indicate statistically significant differences between groups (*P* < 0.05)

### Exploratory Analysis of Factors Associated with Serum P Measured on the Day of Embryo Transfer

According to the univariate analysis, a statistically significant difference was found between the groups divided according to the serum P cutoff in the following variables: type of infertility, transfer of a good-morphology embryo, number of retrieved oocytes, number of mature oocytes recovered, and clinical pregnancy rate. In the multivariate logistic regression model, in turn, only the type of infertility remained statistically different between groups, being the sole independent predictor of serum P concentration on the day of FET in AEP cycles (Table 6).

**Table 6:** Multivariate analysis of predictors of serum P concentration above 10.6 ng/mL on the day of frozen-thawed embryo transfer in artificial endometrial preparation cycles

	Adjusted odds ratio (95% CI)
Secondary infertility	2.5 (1.25 – 5)
Transfer of good-morphology embryo	1.6 (0.9 – 3.3)
No. of retrieved oocytes	0.9 (0.8 – 1)
No. of mature oocytes recovered	1 (0.9 – 1.2)
Clinical pregnancy	2 (0.9 – 5)

Odds ratio estimated using the multivariate logistic regression model, in which the event is coded as 0

## Discussion

There has been much debate about the role of serum P measurement on the day of FET and its association with pregnancy success. The literature remains quite controversial on this topic [7, 8, 13, 14, 20, 28, 32]. Moreover, there is no consensus on the optimal serum P cutoff on the day of FET that would be associated with better pregnancy outcomes [3, 7, 12–14, 19, 28, 31]. Additionally, there is considerable variability in serum P concentrations on the day of FET, which may be due to individual patient characteristics such as obesity and overweight [5], medication bioavailability [23], and the timing of blood sample collection [17], among other factors that warrant further investigation.

Given the uncertainty regarding the potential predictive role of serum P concentration on the day of FET using autologous oocytes in AEP cycles, we conducted the present study. We constructed a ROC curve to identify the serum P cutoff associated with a higher CP rate, identifying a minimum value of 10.6 ng/mL, with a sensitivity of 72%, very low specificity (44%), and a poor AUC (0.58), indicating the test has limited predictive value. Despite our ROC curve being very similar to those found in other published studies [7, 13, 14], the authors approached the issue differently, claiming that serum P concentration on the day of embryo transfer in AEP cycles is a good predictor of pregnancy success.

In 2017, Labarta et al. published a prospective cohort study of oocyte reception cycles in which one or two good-quality blastocysts, either fresh or thawed, were transferred following AEP with oral or transdermal estrogen and 400 mg of vaginal micronized progesterone every 12 h for five days. They suggested that serum P concentration on the day of FET was a predictor of ongoing pregnancy, and that levels below 9.2 ng/mL were associated with lower pregnancy success. However, in the ROC curve they developed, the P cutoff was 11 ng/mL, with an AUC of 0.59, sensitivity of 70%, and specificity of 50.5%. What we observe is that, although the study showed a statistically significant difference in ongoing pregnancy rates depending on serum P values on the day of FET, the ROC curve demonstrated a rather limited test performance due to the low AUC—similar to what was found in the present study.

In 2021, Labarta et al. published another prospective cohort study with the same endometrial preparation protocol but expanding the number of patients and including, in addition to oocyte reception cycles, cycles with blastocysts derived from autologous oocytes, fresh or thawed, with and without PGT-A. In their study, again, a significantly lower ongoing pregnancy rate was observed in patients who had serum P levels on the day of FET below the identified cutoff, which was 8.8 ng/mL. In the ROC curve constructed to identify this cutoff, the AUC was 0.58, with a sensitivity of 77.6% and specificity of 37.1%, again similar to those found in the present study. Nevertheless, in their conclusions, the authors stated that serum P concentration on the day of embryo transfer in AEP cycles is a good predictor of ongoing pregnancy. It is worth noting that the authors jointly evaluated cycles with blastocysts from both fresh and cryopreserved donated oocytes, as well as cycles with blastocysts from autologous oocytes, with or without PGT-A, fresh or thawed, which could be a source of bias. Of the total 1,150 cases evaluated, only 184 were blastocyst transfer cycles derived from autologous oocytes without PGT-A, similar to those evaluated in our study. In this subgroup, ongoing pregnancy rates were not significantly different between patients with serum P concentrations above or below the established cutoff (8.8 ng/mL), similarly to our findings.

Although we initially found a statistically significant reduction in the CP rate in the group of patients with serum P levels on the day of FET below the identified cutoff (10.6 ng/mL), when we performed multivariate logistic regression to exclude confounding factors, this variable was not an independent predictor of this outcome. Corroborating this result, Volovsky et al. conducted a retrospective cohort study in 2020 with 2,010 patients undergoing FET in AEP cycles using 2.0 mg of estradiol (twice daily) and 200 mg of vaginal progesterone (every 8 h), and observed no statistically significant differences in biochemical pregnancy, CP, or live birth rates when patients were divided by a serum P cutoff of 10 ng/mL on the day of FET. However, there was a lower live birth rate in patients with serum P levels below 5.0 ng/mL on the day of FET, although without significant differences in biochemical or clinical pregnancy rates.

Thus, we emphasize that measuring serum P on the day of FET in AEP cycles remains highly uncertain in the literature. The type of preparation cycle, the P supplementation route, and the dosage may impact study outcomes. Kofinas et al. published a retrospective study in 2015 that evaluated serum P on the day of FET and its relationship with pregnancy outcomes in patients who underwent euploid embryo transfer with luteal phase support via intramuscular P. The authors found that patients with serum P levels above 20 ng/mL on the day of FET had lower ongoing pregnancy and live birth rates, and higher rates of miscarriage and biochemical pregnancy. Alyasin et al. published a prospective cohort in 2021 with AEP cycles for FET supplemented with intramuscular and vaginal P, and observed that serum P concentrations above 32.5 ng/mL on the day of FET were associated with lower live birth rates. Their ROC curve for this cutoff had an AUC of 0.61 and specificity of 50%. Melo et al. [21] published a prospective multicenter cohort analyzing the relationship between serum P concentrations on the day of FET and pregnancy outcomes according to endometrial preparation type and route of progestogen administration. They observed that in artificial cycles with vaginal progestogen, live birth rates increased with increasing serum P concentration. However, in artificial cycles with subcutaneous progestogen, live birth rates increased with serum P levels up to 16.3 ng/mL, but decreased beyond that level. Therefore, while some studies find a minimum cutoff for serum P on the day of FET, others find a maximum cutoff, above which poorer pregnancy outcomes are observed. Still, others find no correlation between serum P on the day of FET and pregnancy outcomes. Some studies suggest there might be an ideal range for serum P on the day of FET, where levels that are too low may delay endometrial development, and levels that are too high may accelerate it. However, besides the methodological heterogeneity and inconsistent results across studies, even those showing serum P on the day of FET as a predictor of pregnancy success demonstrate limited predictive power for this variable.

We found no significant difference in serum P concentrations on the day of FET between patients with and without CP. Most of the cited studies did not, as we did, analyze patients divided according to pregnancy outcome to assess whether there was a difference in serum P levels between those who became pregnant and those who did not. These studies only performed statistical analyses dividing patients based on a cutoff point found or defined in the literature.

As a secondary objective, our study evaluated predictors of CP and found that the transfer of blastocyst-stage and of good-morphology embryos were prognostic factors for CP, while the presence of endometriosis was negatively associated with this outcome, corroborating some published studies [4, 11, 16, 27].

Also, as a secondary objective, we investigated which variables predicted serum P levels above 10.6 ng/mL on the day of FET and found that only the presence of secondary infertility was a predictor in our study. Few studies have investigated this objective. In a 2022 observational cohort study [18] evaluating 915 patients undergoing FET with artificial cycles, smoking, high BMI, and multiparity were associated with lower serum P concentrations on the day of FET. This may reflect that other variables should actually be considered when regarding serum P as a predictor of pregnancy, since some variables may be correlated with low serum P on the day of FET and also act as negative predictors for pregnancy success. Thus, the correlation between serum P and the pregnancy outcome might be misleading.

This study has some limitations that should be considered. As an observational analysis, the examined variables were obtained from the patients’ medical records. Although a prospective cohort study was conducted and various efforts were made to reduce bias, the number of participants was relatively small, and the rate of missing serum P measurements on the day of FET was high. Therefore, studies with larger samples are needed to corroborate our findings.

The quantification of P levels was performed only once on the day of FET. Although variations may occur throughout the day due to the endogenous pulsatility of P, a single measurement during the mid-luteal phase can provide meaningful information about the endometrium’s exposure to this hormone. To minimize the effect of circadian variation in endogenous progesterone levels, blood samples were collected within a standardized time window (between 8:00 and 10:00 a.m.). Additionally, in the present study, the same kit and methodology for P measurement were used for all patients to standardize the results. However, our findings cannot be generalized to other methods of serum P measurement.

Another potential limitation is that the transferred embryos were at both the cleavage and blastocyst stages, and the results were not analyzed separately according to embryo developmental stage. However, the majority of transfers involved cleavage-stage embryos.

There remain many gaps in knowledge regarding serum P measured on the day of FET as a predictor of pregnancy success. The type of endometrial preparation cycle, the route and dosage of progesterone supplementation during the luteal phase, as well as the outcome analyzed, are factors that potentially influence the conflicting results reported in the literature. Moreover, there are factors that independently influence serum P concentrations and CP rates, which may act as confounders in analyzing the relationship between serum P on the day of FET and the pregnancy outcome. For patients undergoing FET using autologous oocytes without PGT-A in an AEP cycle with estrogen and vaginal P, we observed that serum P concentration on the day of FET has limited validity as a predictive test for CP.

## Conclusion

In conclusion, for frozen-thawed embryo transfer cycles using autologous oocytes without PGT-A, with artificial endometrial preparation cycles using estrogen and vaginal progesterone at a dose of 600 mg/day, we noted that serum P concentration on the day of FET is not a good predictor of clinical pregnancy. If future studies with larger sample sizes and transfers restricted to embryos at a single developmental stage confirm our findings, there would be no justification for routinely measuring serum P on the day of FET to predict clinical pregnancy or to guide clinical decisions such as canceling the transfer or adding alternative routes of P administration.

## Data Availability

All data reported are available from the corresponding author upon reasonable request.
